# Colchicine overdose impairs the capacity of Kupffer cells to clear foreign particles and endotoxins

**DOI:** 10.1007/s00204-022-03353-8

**Published:** 2022-09-14

**Authors:** Reham Hassan, Maiju Myllys, Lisa Brackhagen, Zaynab Hobloss, Daniela González, Abdel-latif Seddek, Adrian Friebel, Stefan Hoehme, Rosemarie Marchan, Michael Trauner, Jan G. Hengstler, Ahmed Ghallab

**Affiliations:** 1 Leibniz Research Centre for Working Environment and Human Factors, Technical University Dortmund, Ardeystr. 67, 44139 Dortmund, Germany; 2 Department of Forensic Medicine and Toxicology, Faculty of Veterinary Medicine, South Valley University, Qena, 83523 Egypt; 3 Institute of Computer Science and Saxonian Incubator for Clinical Research (SIKT), University of Leipzig, Haertelstraße 16-18, 04107 Leipzig, Germany; 4 Hans Popper Laboratory of Molecular Hepatology, Division of Gastroenterology and Hepatology, Department of Internal Medicine III, Medical University of Vienna, Vienna, Austria

**Keywords:** Intravital imaging, Microtubules, Lipopolysaccharide, Drug toxicity, Phagocytosis

## Abstract

Colchicine is an anti-inflammatory drug with a narrow therapeutic index. Its binding to tubulin prevents microtubule polymerization; however, little is known about how depolymerization of microtubules interferes with the phagocytosis function of Kupffer cells (KC). Here, we applied functional intravital imaging techniques to investigate the influence of microtubule disruption by colchicine on KC morphology, as well as its capacity to clear foreign particles and bacterial lipopolysaccharide (LPS) in anesthetized mice. Intravital imaging of KC in healthy mice showed the typical elongated morphology, localization at the luminal side of the sinusoidal endothelial cells, and moving cell protrusions. In contrast, at colchicine doses of 1 mg/kg and higher (intraperitoneal), KC appeared roundish with strongly reduced protrusions and motility. To study the functional consequences of these alterations, we analyzed the capacity of KC to phagocytose fluorescent nanospheres (100 nm-size) and LPS. After tail vein injection, the nanospheres formed aggregates of up to ~ 5 µm moving along the sinusoidal bloodstream. In controls, the nanosphere aggregates were rapidly captured by the Kupffer cell protrusions, followed by an internalization process that lasted up to 10 min. Similar capture events and internalization processes were observed after the administration of fluorescently labeled LPS. In contrast, capture and internalization of both nanospheres and LPS by KC were strongly reduced in colchicine-treated mice. Reduced phagocytosis of LPS was accompanied by aggravated production of inflammatory cytokines. Since 0.4 mg/kg colchicine in mice has been reported to be bio-equivalent to human therapeutic doses, the here-observed adverse effects on KC occurred at doses only slightly above those used clinically, and may be critical for patients with endotoxemia due to a leaky gut–blood barrier.

**Supplementary Information:**

The online version contains supplementary material available at 10.1007/s00204-022-03353-8.

## Introduction

Colchicine, an alkaloid produced by the poisonous plant *Colchicum autumnale*, is one of the oldest available drugs, and still is widely used for the treatment of inflammatory diseases, including gout, osteoarthritis, familial Mediterranean fever, pericarditis, and Behcet’s disease (Leung et al. 2015). It binds to both alpha- and beta-tubulin, which prevents the formation of microtubules (Dalbeth et al. 2014), reduces the migratory capacity of cells, and decreases the recruitment of myeloid cells to inflamed tissues (Dinarello et al. 1976; Weng et al. 2021). However, colchicine has a relatively narrow therapeutic index; although there is no clear cut between therapeutic and toxic doses, administration of 0.5 mg/kg or higher doses is generally considered as toxic (Finkelstein et al. 2010). Even at therapeutic doses, colchicine may compromise physiological immune functions, which, for example, may increase the risk of pneumonia in gout patients who were treated with colchicine, compared to those who did not use this drug (Tsai et al. 2019).

Kupffer cells (KC), the macrophages of the liver, represent the largest population of resident tissue macrophages in the body (Dixon et al. 2013). Their strategic localization at the blood side of the sinusoidal endothelial cells allows them to efficiently phagocytose foreign particles and bacterial lipopolysaccharide (LPS) from the sinusoidal blood that may pass from the gastrointestinal tract via the portal circulation. LPS is an efficient alarm molecule that is sensed by innate immune cells (Opal 2010), and in small quantities, it induces an anti-microbial defense. However, if LPS suddenly exceeds critical thresholds, it may cause an imbalanced overproduction of proinflammatory cytokines, also named cytokine storm, which is deleterious to the host as it may lead to septic shock (Gustot et al. 2009; Opal 2010).

Currently, it is unknown if therapeutic doses of colchicine compromise the capacity of KC to clear the blood of foreign particles. The direct analysis of Kupffer cell function has been difficult. However, the recent establishment of functional intravital imaging techniques based on two-photon microscopy has made it possible to directly observe parenchymal and non-parenchymal cells in intact livers of anesthetized mice at subcellular resolution (Brecklinghaus et al. 2022; Ghallab et al. 2022, 2019a; Hassan 2016; Schneider et al. 2021a). KC can be visualized intravitally after intravenous injection of fluorophore-coupled antibodies or using cell-type specific reporter mice (Reif et al. 2017; Remetic et al. 2022). Recording of sequences in the millisecond range allows for the direct analysis of fast processes (Koppert et al. 2018; Vartak et al. 2021), such as capture events of particles from liver sinusoidal blood by KC. In the present study, we report that colchicine doses, which are only slightly above those applied therapeutically, strongly reduce the capacity of KC to clear foreign particles and LPS from the blood.

## Materials and methods

### Mice, colchicine treatment, and sample collection

Eight-to-ten-week-old male C57BL6/N mice were used (Janvier Labs, France), as well as the macrophage reporter LysM-Cre mice (Clausen et al. 1999) after breeding with the Cre-reporter mT/mG mice (Muzumdar et al. 2007) (The Jackson Laboratory, USA). All mice were housed under standard conditions (Gianmoena et al. 2021), with ad libitum feeding on a normal rodent diet (V1534-000, Ssniff) and free access to drinking water. The experiments were approved by the local animal ethics committee. Colchicine (#C9754, Sigma-Aldrich) was dissolved in phosphate-buffered saline (PBS) and was administered intraperitoneally in C57BL6/N mice at doses of 0, 0.25, 0.5, 1, 2, or 4 mg/kg. The application volume was 4 ml/kg. Four mice were used per group. Six hours after colchicine injection, the mice were anesthetized and blood samples were collected from the heart in syringes pre-coated with ethylenediamine tetra-acetic acid (EDTA), as previously described (Ghallab et al. 2019b). Subsequently, the liver was excised and a piece of approximately 5 × 7 mm was taken from the left liver lobe and fixed in 4% paraformaldehyde (Roti-Histofix, #P 087–5, Roth) for 2 days, washed in PBS, and finally embedded in paraffin to be used for immunohistochemistry (Campos et al. 2020).

### Clinical chemistry

Plasma levels of alanine transaminase (ALT), aspartate transaminase (AST), alkaline phosphatase (ALP) activities, and glucose concentration were determined using the Piccolo Xpress Chemistry Analyzer (Hitado, Germany).

### Immunohistochemistry

Immunohistochemistry was performed in 4 µm-thick paraffin-embedded liver tissue sections using an autostainer (Discovery Ultra Automated Slide Preparation System, Roche, Germany), as previously described (Ghallab et al. 2021b; Holland et al. 2022). Anti-alpha-tubulin (#2144, Cell Signaling), anti-F4/80 (#MCA497, Bio-Rad), and anti-cleaved caspase-3 (#9661S, Cell Signaling) primary antibodies were used for staining of microtubules, macrophages, and apoptotic cells, respectively (Table 1). Appropriate Ultra-Map anti-rabbit or anti-rat HRP secondary antibodies (Roche, Germany) were used (Table 1).

**Table 1:** Antibodies and functional dyes used for immunohistochemistry and intravital imaging

Target	Primary antibodies		Secondary antibodies	
	Antibody	Dilution	Antibody	Dilution
*Antibodies used for immunohistochemistry*				
Microtubules	Anti-alpha-tubulin	1:50	Ultra-Map anti-rabbit HRP	Automatic discovery ready to use
Macrophages	F4/80	1:100	Ultra-Map anti-rat HRP	
Apoptosis	Cleaved caspase-3	1:500	Ultra-Map anti-rabbit HRP	

Dye/marker/reporter	Marker for	Dose	Vehicle	Excitation range (nm)
*Functional dyes, reporter mice, and antibodies used for intravital imaging*				
Rhodamine 123	Mitochondrial membrane potential	0.8 mg/kg	Methanol:PBS (1:1)	720–820
PE-F4/80 antibody	Macrophages	0.06 mg/kg	PBS	720–760
Alexa Fluor594-F4/80 antibody		0.06 mg/kg	PBS	780–820
LysM reporter mouse		–	–	800–860
Hoechst 33258	DNA	5 mg/kg	PBS	720–840
FluoSpheres Carboxylate-modified nanospheres	Macrophage uptake capacity	40 µl/mouse	–	720–820
LPS-Alexa Fluor 488	LPS clearance	3 mg/kg	PBS	740–780
Cholyl-lysyl-fluorescein	Bile acid analogue	1 mg/kg	PBS	740–820

### Intravital imaging and image analysis

Intravital imaging of intact livers was done in anesthetized mice using a two-photon microscope (LSM MP7, Zeiss, Germany), as previously described (Ghallab et al. 2022; Schneider et al. 2021a). Mitochondrial potential, macrophages, and cell nuclei were visualized by intravenous administration of the functional dye Rhodamine 123 (#R302, ThermoFisher Scientific), a fluorophore-coupled anti-F4/80 antibody (#15-4801-82, ThermoFisher Scientific), and Hoechst 33,258 (#H2149, ThermoFisher Scientific), respectively, before recording (Table 1) (Reif et al. 2017). To determine the uptake capacity of KC, 40 µl of fluorescent carboxylate-modified nanospheres, 100 nm-size (#F8803, ThermoFisher Scientific), were administered via a tail vein catheter while recording (SAI-infusion, IL, USA). To record lipopolysaccharide (LPS) clearance, LPS-Alexa Fluor 488 conjugate from *Escherichia Coli* serotype O55:B5 (#L23351, ThermoFisher Scientific) was administered while recording via a tail vein catheter (Remetic et al. 2022). To visualize bile canaliculi, a bolus of the bile acid analogue cholyl-lysyl-fluorescein (CLF; #451041, Corning) was intravenously injected via a tail vein catheter approximately 15 min before the end of the recording (Vartak et al. 2021). Image analysis was done in defined regions of interest using Zen software (Zeiss, Germany), as previously described (Ghallab et al. 2021a; Koeppert et al. 2021). Representative video/image of at least 4 mice per condition was analyzed and presented in the result section.

### LPS challenge and cytokine assay

To investigate whether colchicine toxicity aggravates the production of inflammatory cytokines after LPS challenges, male C57Bl6/N mice were treated with colchicine (2 mg/kg) or PBS; and 6 h later, the mice were challenged intravenously with 4 mg/kg LPS from *Escherichia Coli* serotype O55:B5 (#L2880, Sigma-Aldrich) (Schneider et al. 2021b). Four mice were used per group. Blood samples were collected before (0 h) as well as at 1 and 2 h after LPS injection. Plasma levels of the proinflammatory cytokines TNF-α (#MTA00B, R&D Systems) and IL6 (#M6000B, R&D Systems) were detected by enzyme-linked immunosorbent assays (ELISAs) according to the manufacturer’s protocols.

### Statistical analysis

Data were statistically analyzed using the GraphPad Prism software version 9.3.1 (GraphPad Software, Inc., La Jolla, CA, USA). Dunnett's and Tukey's multiple comparisons tests were used as indicated in the figure legend. The data are expressed as mean ± SE.

## Results

### Dose-dependent hepatotoxicity of colchicine

**Fig. 1:**
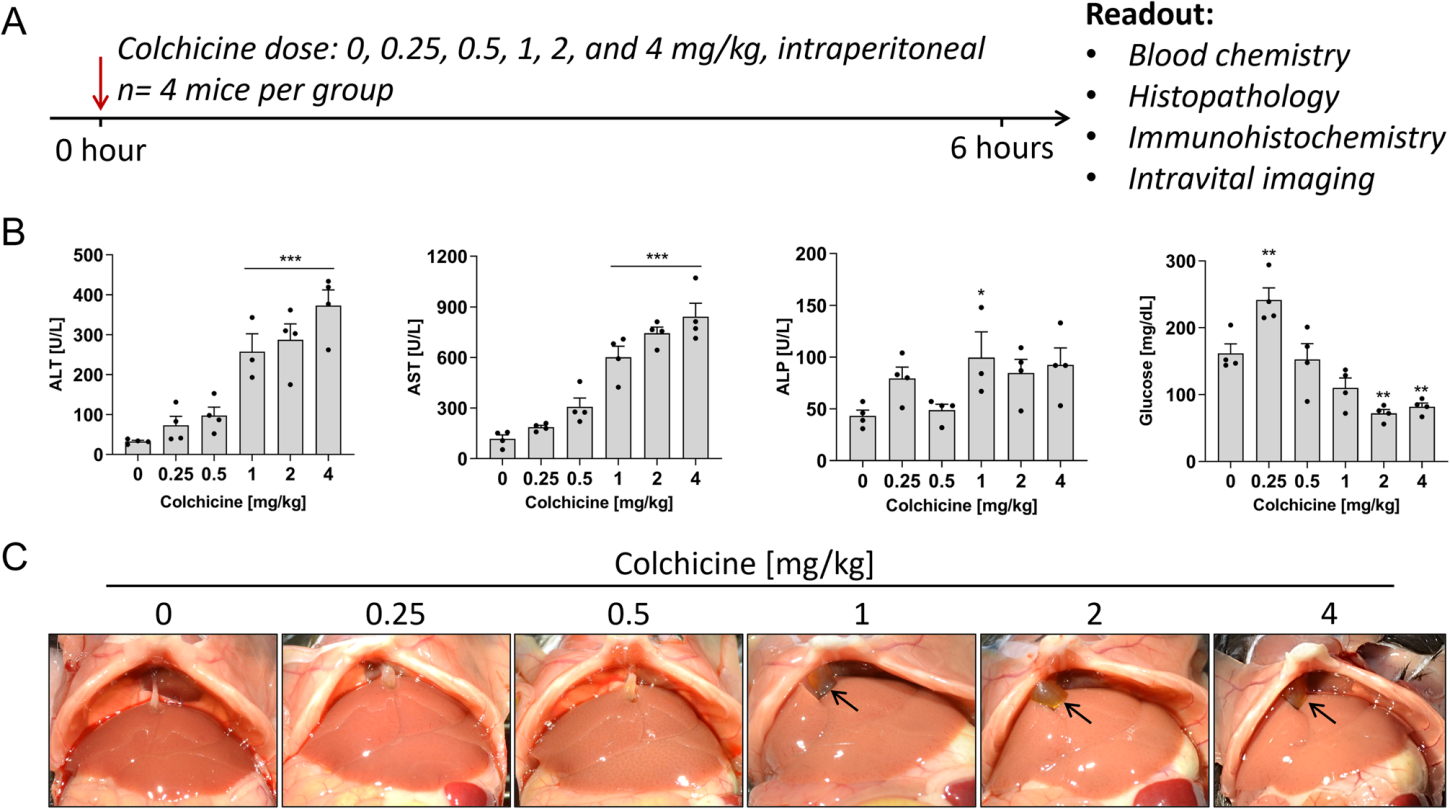
Dose-dependent hepatotoxicity of colchicine. **A** Experimental design; *n* = 4 mice per group. **B** Plasma levels of alanine transaminase (ALT), aspartate transaminase (AST), and alkaline phosphatase (ALP) activities and glucose concentrations 6 h after administration of different doses of colchicine; *p* < 0.05 (*), *p* < 0.01 (**), and *p* < 0.001 (***), Dunnett's multiple comparisons test. Data of individual mice are illustrated by dots. **C** Gross pathology of the liver showing dose-dependent pale discoloration, and bile accumulation in the gallbladder (arrows) at 1 mg/kg and higher doses of colchicine.

Colchicine was dose-dependently administered to mice to determine the activity of liver enzymes in blood (Fig. 1A). A dose-dependent increase in the hepatocellular damage markers, ALT and AST, was observed over the entire dose range of 0.25–4 mg/kg 6 h after administration, with statistically significant differences compared to vehicle controls for the three highest doses of 1, 2, and 4 mg/kg (Fig. 1B). In contrast, the bile duct injury and cholestasis marker ALP was not significantly elevated for all tested doses except for the 1 mg/kg dose (Fig. 1B). Blood glucose increased at 0.25 mg/kg and then decreased at higher doses (Fig. 1B). The gross pathology showed dose-dependent pale discoloration of the liver after colchicine treatment, and bile accumulation in the gallbladder after the administration of 1 mg/kg and higher colchicine doses (Fig. 1C).

### Cell-type specific inhibition of microtubule assembly by colchicine

**Fig. 2:**
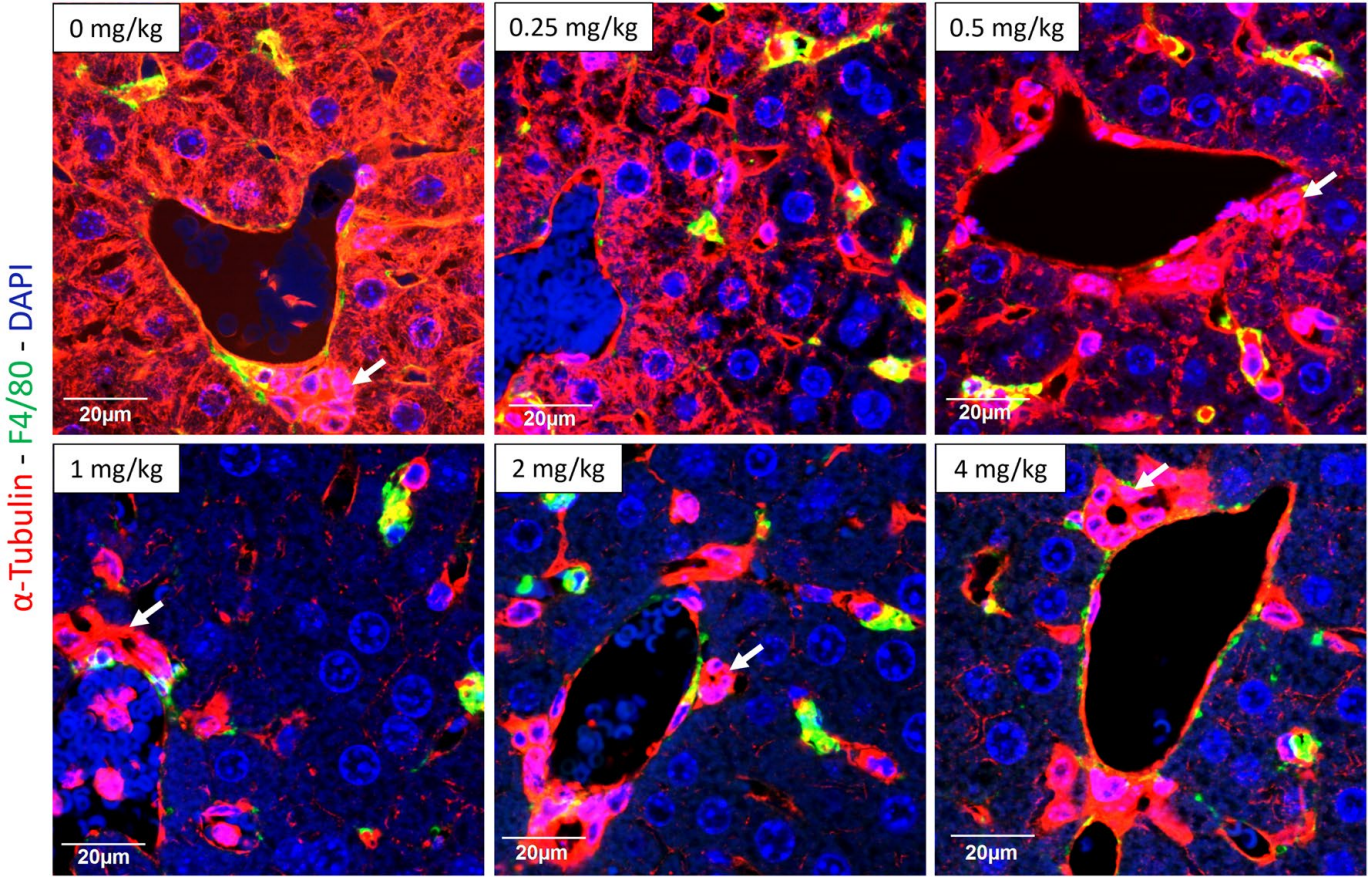
Cell-specific microtubule depletion in the liver after administration of different doses of colchicine. At doses of 0.25 and 0.5 mg/kg, colchicine targets the microtubules of mainly hepatocytes (seen with decreasing red signal in hepatocytes); at doses of 1 mg/kg and higher, it additionally depletes microtubules of Kupffer cells (visualized by F4/80 staining in green). In contrast, microtubules of cholangiocytes (arrows) and endothelial cells of the portal vein branches were not affected with all tested doses of colchicine. Scale bars: 20 µm. Representative images of 4 mice per group are shown

**Fig. 3:**
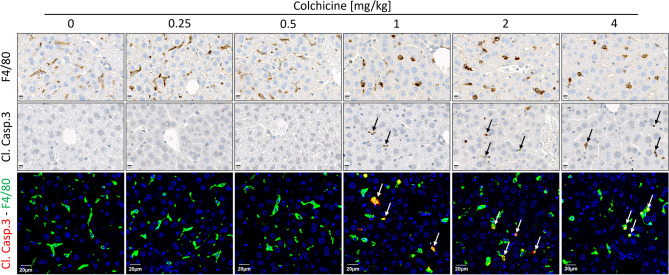
Compromised morphology and apoptosis of Kupffer cells after the administration of high doses of colchicine. F4/80 staining shows the normal elongated Kupffer cell morphology in controls (0 mg/kg) and after administration of low doses of colchicine (0.25 and 0.5 mg/kg); at higher colchicine doses (≥ 1 mg/kg), Kupffer cells appear rounded and undergo apoptosis as evidenced by cleaved caspase-3 staining (arrows). Scale bars: 10 µm (F4/80 and cleaved caspase-3 staining); 20 µm (co-staining of F4/80 and cleaved caspase-3). Representative images of 4 mice per group are shown

To study the influence of colchicine on microtubules in the liver tissue of mice, formalin-fixed paraffin-embedded liver tissue sections were immunostained using antibodies directed against α-tubulin and the macrophage marker F4/80. Under these conditions, KC and—based on their specific morphology—cholangiocytes, hepatocytes, and the endothelial cells of the portal vein branches could be differentiated (Fig. 2). Interestingly, susceptibility to colchicine-induced microtubule depletion differed among the cell types. Hepatocytes represented a particularly susceptible cell type with reduced α-tubulin signal observed already at 0.25 and 0.5 mg/kg, which decreased even more strongly at 1 mg/kg and higher doses (Fig. 2). In contrast, cholangiocytes and the endothelial cells of the portal vein showed no reduction in the α-tubulin signal up to the highest tested dose of 4 mg/kg (Fig. 2). KC were analyzed by immunostaining with green-fluorescent anti-F4/80 and red-fluorescent anti-α-tubulin antibodies. Since F4/80 is predominantly localized to the cell surface, this resulted in a yellow cell margin and red cytoplasm for the controls (Fig. 2), a pattern which remained relatively constant after the administration of 0.25 and 0.5 mg/kg colchicine. However, after administering the higher doses (1, 2, and 4 mg/kg), the tubulin-associated red fluorescence of the cytoplasm was lost, with the result that KC predominantly appeared green and yellow (Fig. 2). Next, the influence of colchicine on Kupffer cell morphology was further studied by immunoperoxidase staining using antibodies against F4/80. In the control mice and after administration of low doses of colchicine (0.25 and 0.5 mg/kg), KC exhibited their normal, elongated morphology which became more rounded after the administration of 1 mg/kg and higher doses (Fig. 3). The morphological alteration at doses of 1 mg/kg and higher was accompanied by an increase in the signal of cleaved caspase-3 in some cells (Fig. 3). To study if the caspase-3 positive cells were the KC, we performed co-immunostaining of F4/80 (green) and cleaved caspase-3 (red). Co-localization (yellow) demonstrated that indeed a fraction of the KC became caspase-3 positive at colchicine doses of 1 mg/kg and higher (Fig. 3).

### Colchicine compromises the ability of Kupffer cells to phagocytose nanoparticles and LPS

**Fig. 4:**
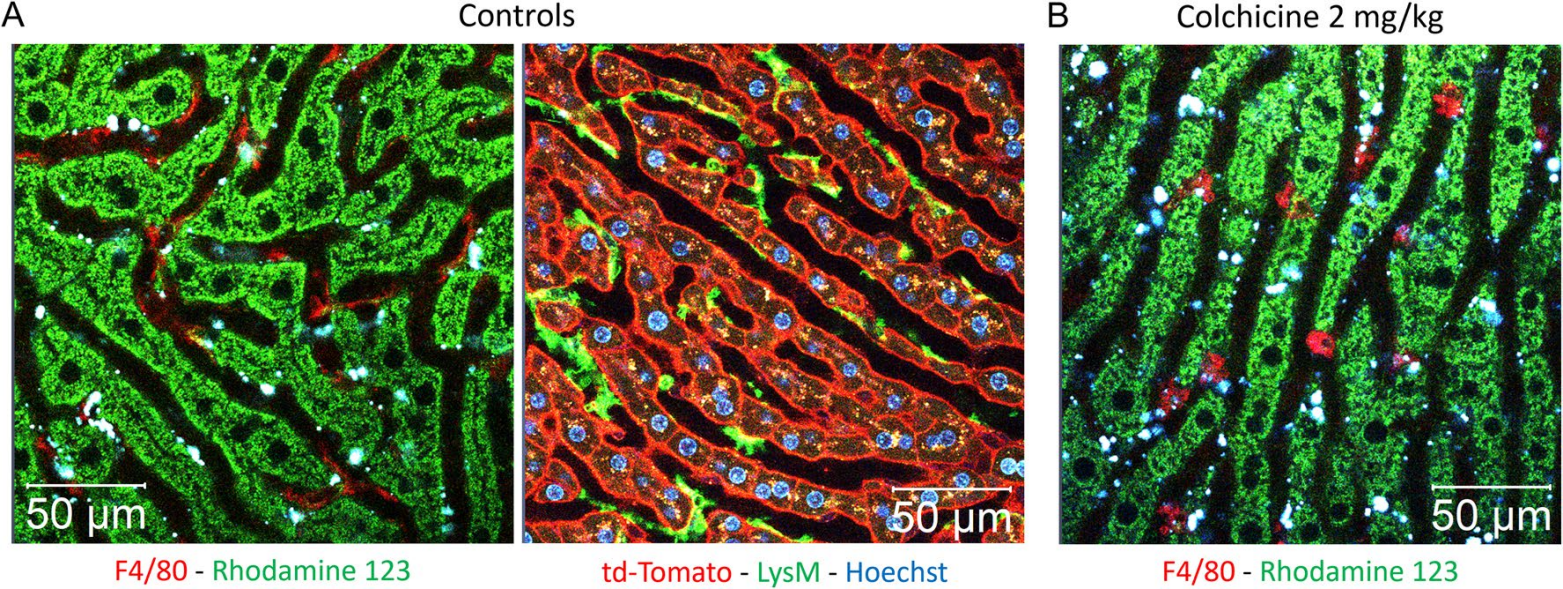
Intravital imaging of Kupffer cells in control and in colchicine-intoxicated mice. **A** Stills from intravitally recorded videos in control mice showing elongated Kupffer cells with irregular surface and numerous protrusions. The Kupffer cells are visualized by intravenous administration of a red-fluorescent antibody (left; corresponds to supplemental video 1A) or using the macrophage reporter mouse LysM (right; corresponds to supplemental video 1B). **B** A still of intravitally recorded video 6 h after colchicine intoxication showing rounded Kupffer cells with smooth surface and no protrusions; the Kupffer cells are visualized by intravenous administration of a red-fluorescent antibody (corresponds to supplemental video 1C). Scale bars: 50 µm. Representative images of 4 mice per group are shown

To functionally characterize the influence of colchicine on KC in intact livers of anesthetized mice, we applied two-photon-based intravital imaging (Reif et al. 2017). Using this intravital imaging toolbox, KC in vivo can be visualized by the intravenous injection of a fluorophore-coupled anti-F4/80 or by lysozyme M (LysM) mediated GFP expression in td-tomato mice (Fig. 4). In agreement with the immunostaining data, intravital imaging confirmed the transition of KC from an elongated (Fig. 4A) to a roundish (Fig. 4B) morphology after colchicine exposure. Moreover, the corresponding time-lapse videos (Supplemental Video 1) demonstrated that KC in control mice formed protrusions into the lumen of the sinusoidal blood capillaries which showed highly active motility, in contrast to the static cell body (Supplemental Videos 1A, B). However, the roundish KC failed to form the protrusions after colchicine exposure and were much more static (Supplemental Video 1C).

**Fig. 5:**
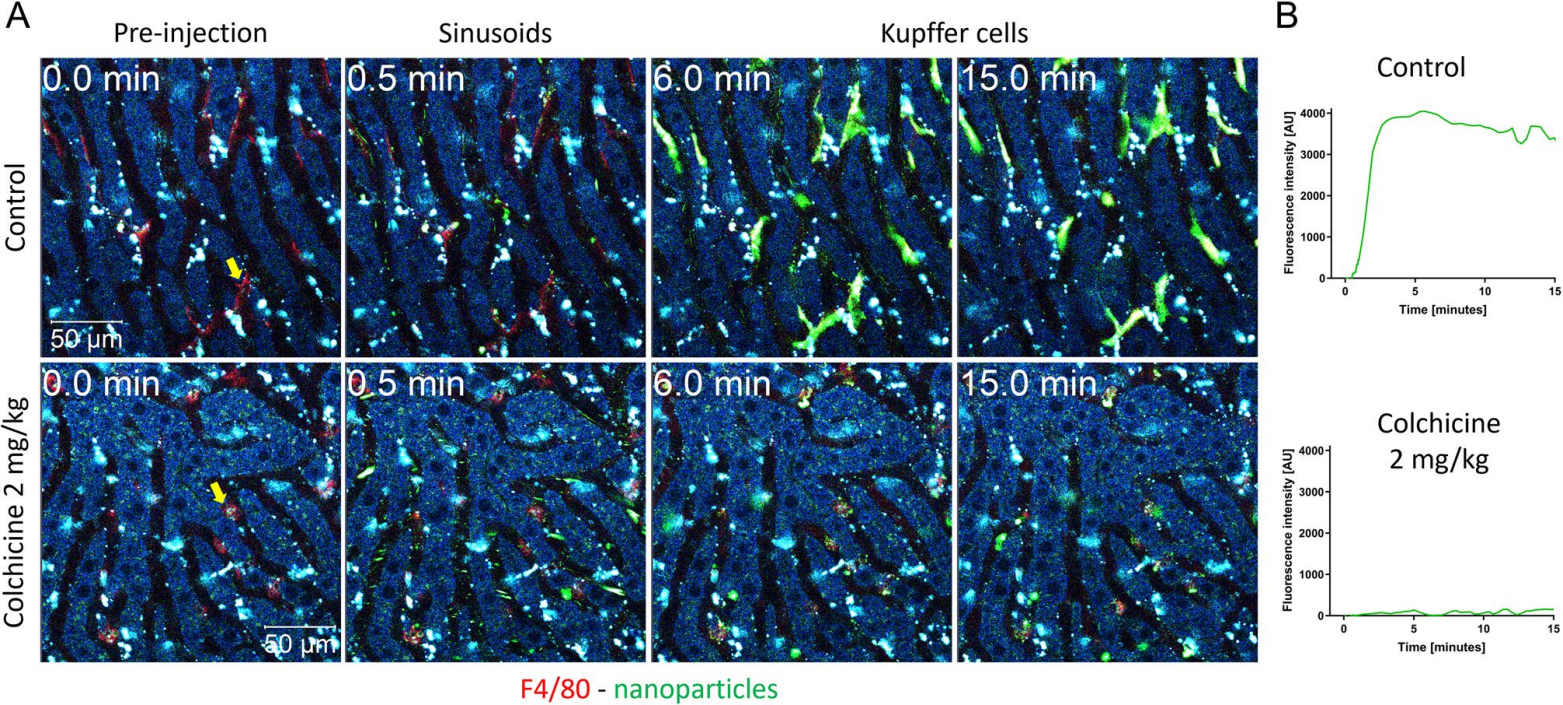
Compromised nanoparticle uptake capacity of macrophages after colchicine intoxication. **A** Stills from intravitally recorded videos in control (upper panel; corresponds to Supplemental video 2A) and in colchicine-intoxicated (lower panel; corresponds to Supplemental video 2B) mice, before (0 min) and at different time intervals after intravenous administration of green-fluorescent 100 nm-size nanoparticles. The images show rapid uptake of the nanoparticles by Kupffer cells (visualized by red-fluorescent F4/80 antibody) in the control mouse which becomes green. In contrast, Kupffer cells of the colchicine-intoxicated mouse failed to take up the nanoparticles. Scale bars: 50 µm. **B** Quantification of the nanoparticle signal intensity in representative Kupffer cells of control and colchicine-treated mice (indicated by the arrows in **A**). Representative images of 4 mice per group are shown

To analyze the influence of these alterations on phagocytic capacity of KC, green-fluorescent 100 nm-size nanospheres that are known to be phagocytosed by macrophages (Reif et al. 2017) were injected into controls and colchicine-treated mice via a tail vein catheter. KC were visualized by a red-fluorescent F4/80 antibody injected intravenously before recording. In the control mice, the initially red-fluorescent KC rapidly adopted a green color due to the uptake of the nanospheres (Fig. 5; Supplemental video 2A). In contrast, the roundish KC in colchicine-treated mice showed reduced uptake of nanospheres (Fig. 5; Supplemental Video 2B). The very strong inhibitory effect of colchicine was further demonstrated by the quantification of the nanosphere-associated green fluorescence intensity of the KC (Fig. 5B).

**Fig. 6:**
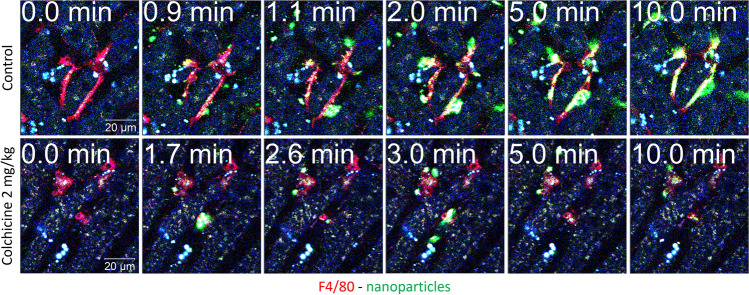
Functional intravital recording of the nanoparticle uptake process by Kupffer cells of healthy and colchicine-intoxicated mice. Closeup of Kupffer cells (visualized by red-fluorescent F4/80 antibody) in control (upper panel; corresponds to Supplemental video 3A) and in colchicine-intoxicated (lower panel; corresponds to Supplemental video 3B) mice, before (0 min) and at different time intervals after intravenous administration of green-fluorescent 100 nm-size nanoparticles. In control mice, the green-fluorescent nanoparticles are captured by the Kupffer cell protrusions, followed by rapid engulfment into the cell body. In contrast, after colchicine intoxication, the nanoparticles transiently stick to Kupffer cells, which fail to engulf them, and then are transported away by the bloodstream. Scale bars: 20 µm. Representative images of 4 mice per group are shown

To visualize the process of phagocytosis, fast sequences with 30 images per minute were recorded at high resolution. The video (Supplemental Video 3A) and corresponding stills of a control mouse show aggregates of particles moving with the sinusoidal blood stream. Between 0.9 and 1.1 min, aggregates are attacked and held by the protrusions at different sites of a Kupffer cell. These capture events are followed by the phagocytic uptake of the captured particles, a process that may take several minutes (for example from min 1.1–10 in Fig. 6 and Supplemental Video 3A). The corresponding Supplemental Video 3A shows a rapid capture event at 0.7–0.9 min followed by the relatively slow uptake, which results in the cell cytoplasm gradually becoming green. This is in contrast to the situation after colchicine treatment, since most nanosphere aggregates simply pass the KC (Supplemental Video 3B; Fig. 6). Capture events were observed only rarely, for example at minutes 1.7 and 3.0; and in these cases, the aggregates were only transiently detained by the KC and subsequently released again into the bloodstream without being phagocytosed (Fig. 6; Supplemental Video 3B).

**Fig. 7:**
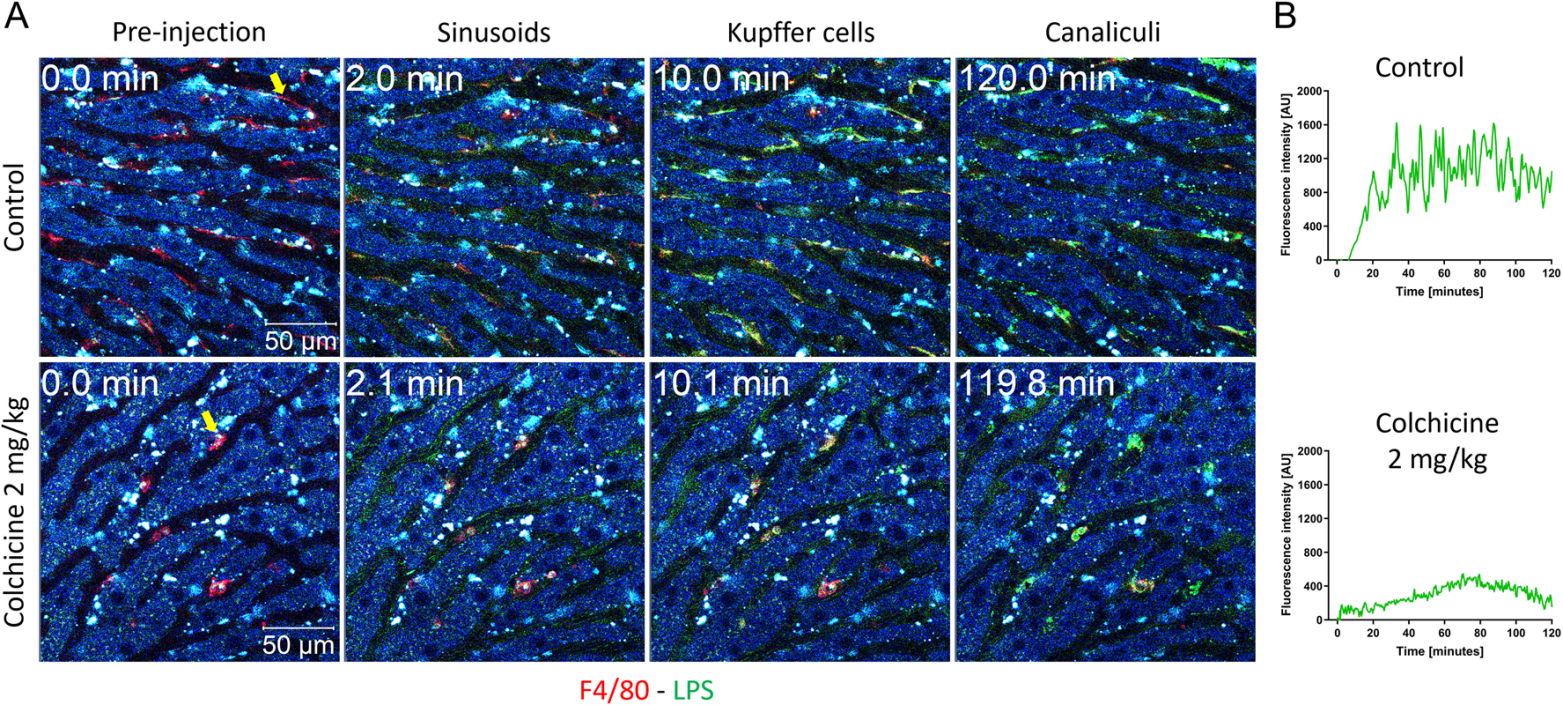
Compromised LPS clearance by Kupffer cells after colchicine intoxication. **A** Stills from intravitally recorded videos in control (upper panel; corresponds to Supplemental video 4A) and in colchicine-intoxicated (lower panel; corresponds to Supplemental video 4B) mice, before (0 min) and at different time intervals after intravenous administration of green-fluorescent LPS. In the control mouse, LPS is rapidly taken up by Kupffer cells (visualized by red-fluorescent F4/80 antibody). In contrast, after colchicine intoxication, the uptake of LPS by Kupffer cells is less and slower. Scale bars: 50 µm. **B** Quantification of the LPS signal intensity in representative Kupffer cells of control and colchicine-treated mice (indicated by the arrows in **A**). Representative images of 4 mice per group are shown

To analyze LPS clearance, we used the fluorophore-labeled LPS-Alexa-Fluor488 in the same experimental setting as described above for the nanospheres. KC of the control mice efficiently internalized LPS-Alexa-Fluor 488 as evidenced by enrichment of the green color in the cell cytoplasm within 5 min after administration (Supplemental Video 4A; Fig. 7). In addition, slow enrichment of LPS in sinusoidal endothelial cells and bile canaliculi was observed (Supplemental Video 4A). In contrast, the uptake of LPS by KC in colchicine-treated mice occurs more slowly and to a lesser degree (Supplemental Video 4B; Fig. 7). Although the difference in LPS clearance by KC between colchicine-treated and control mice is quite obvious (Fig. 7B), it was not as extreme as for the nanospheres (Fig. 5B), which may be explained by the smaller size of the LPS aggregates compared to that of the latter. Similarly, biliary clearance of LPS was blocked by colchicine treatment (Supplemental Video 4B). However, LPS uptake by the sinusoidal endothelial cells was not affected by colchicine intoxication (Supplemental Video 4B).

### Compromised LPS clearance by Kupffer cells aggravates proinflammatory cytokine production

**Fig. 8:**
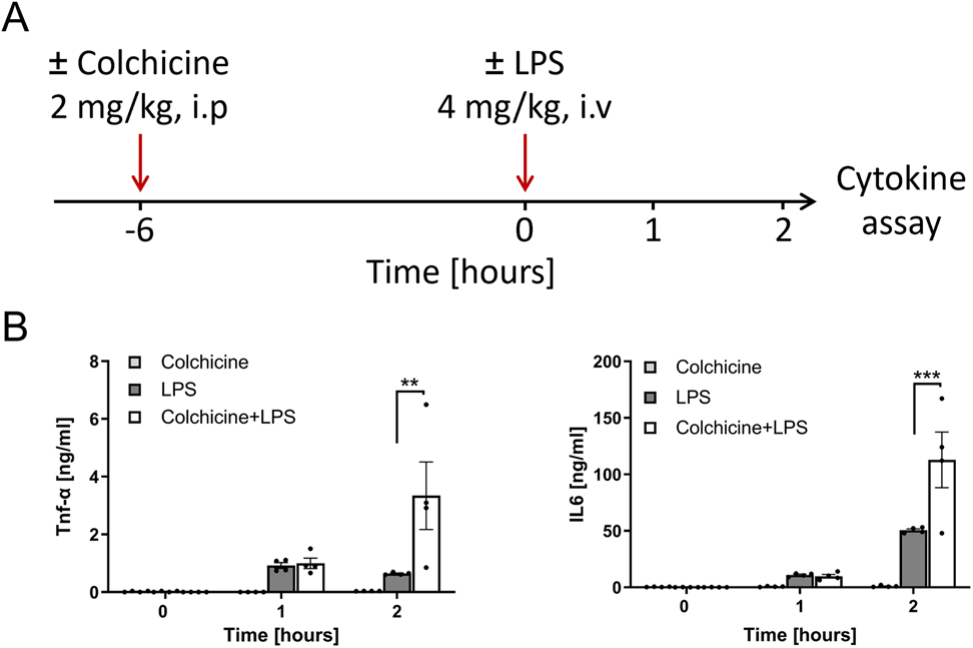
Enhanced cytokine storm in colchicine-intoxicated mice after LPS challenge. **A** Experimental design; *n* = 4 mice per group. **B** TNF-alpha and IL6 levels in plasma at different time intervals after colchicine intoxication, LPS intoxication, and LPS + colchicine intoxication. *p* < 0.01 (**) and *p* < 0.001 (***), Tukey's multiple comparisons test; data of individual mice are illustrated by dots

Since colchicine treatment compromised the clearance of LPS from liver sinusoidal blood by KC, we next studied possible consequences on proinflammatory cytokine levels. For this purpose, mice were pretreated with colchicine (2 mg/kg) or vehicle for 6 h, followed by intravenous application of LPS. Subsequently, plasma levels of the proinflammatory cytokines TNF-α and IL6 were quantified time-dependently after LPS administration by ELISA (Fig. 8A). At 1 h after LPS injection, plasma concentrations of TNF-α and IL6 increased similarly in both the colchicine and vehicle pretreated mice; however, at 2 h after LPS injection, the levels of both cytokines were significantly higher in the colchicine compared to vehicle pretreated mice, leading to ~4-fold (TNF-α) and ~2.5-fold (IL6) higher levels (Fig. 8B). Thus, the compromised LPS clearance capacity of KC was associated with aggravated proinflammatory cytokine production.

## Discussion

In the present study, we demonstrate that colchicine may compromise the clearance of particles and LPS by KC from sinusoidal blood. Based on intravital two-photon microscopy, it was possible to directly observe how KC phagocytose particles in the sinusoidal capillaries. In healthy mice, KC form highly motile protrusions into the sinusoidal lumen that capture moving particles in the bloodstream. These extracellular capture events occur almost instantly. The relatively large size of some of the captured particles, which amounted to ~ 5 µm, may appear surprising, but it should be considered that the here-applied nanospheres of 100 nm-size tend to aggregate in the blood. This is advantageous for intravital imaging, because the size of the formed aggregates is larger than our resolution limit of ~ 200 nm, and as a result, their fate can be directly observed.

Remarkably, KC can capture particles that have much larger diameters than the macrophages themselves. The capture event is followed by internalization of the trapped particles, a process that may last several minutes. Internalization generally takes place at the position of the capture event, and due to the elongated morphology of the KC, it can be observed that the internalized material then moves intracellularly to both sides of the cell. Interestingly, although the Kupffer cell phagocytoses a particle aggregate several-fold larger than its own diameter, there are no major changes to its morphology. This suggests that the captured large nanosphere aggregate is phagocytosed in smaller bite-sized portions, akin to a tiger biting off pieces of its prey rather than the snake analogy, where the prey is swallowed in its entirety. This finding is also consistent with the observation that the extracellular fraction of the nanosphere aggregate gradually becomes smaller over minutes until it is eventually completely transferred into the intracellular space. In addition to the large aggregates (~ 5 µm), smaller particles of less than 0.5 µm were also observed, which were more rapidly internalized than the larger aggregates. Similar capture events and internalization processes were also detected after the administration of fluorescently labeled LPS, but the internalization process occurred much faster, perhaps because of the smaller size of the LPS aggregates. Treatment of mice with colchicine strongly reduced the ability of KC to phagocytose nanospheres and LPS. Even if some capture events of nanosphere aggregates were observed, they did not lead to internalization; rather, the aggregates became detached once more and were carried away by the bloodstream.

Therapeutic doses of colchicine range between 1.2 and 2.4 mg/day for patients with familial Mediterranean fever, 1.2 mg/day in acute gout and 1 mg/day in older studies as antifibrotic drug in liver cirrhosis (Finkelstein et al. 2010; Leung et al. 2015). Allometric scaling and measurement of diarrhea, the dose-limiting toxicity in humans, resulted in 0.4 mg/kg (i.p.), which represents a dose in mice that is bio-equivalent to human clinical doses (Weng et al. 2021). In the present study, we performed a range finding test of doses between 0.25 and 4 mg/kg and observed strong effects on KC with doses of 1 mg/kg and higher. A limitation of the present study is that the translational relevance of the observations made in mice is still difficult to assess in humans (Gupta et al. 2021). It remains to be studied if therapeutic doses in humans also reduce the capacity of KC to clear particles and LPS from the blood. To investigate the possible influence in humans, LPS levels could be analyzed in blood before and after colchicine administration. This may be particularly relevant for individuals with a compromised gut–blood barrier, for example in chronic liver diseases, where the basal levels of LPS are already increased.

Bacterial LPS is a potent mediator of sepsis and septic shock (Opal 2010). A sudden increase of LPS in the bloodstream may cause an extreme release of inflammatory cytokines that generate endothelial injury, disseminated intravascular coagulation, and shock (Opal 2010). The gut–blood barrier may be compromised in liver diseases (Fukui 2021) and patients with liver cirrhosis have an increased risk of developing sepsis accompanied by an imbalanced cytokine response (Gustot et al. 2009). In this scenario, the administration of colchicine, although beneficial to ameliorate inflammation due to the inhibition of macrophage infiltration (Leung et al. 2015), may reduce clearance by KC thereby increasing LPS levels beyond critical thresholds. This scenario would correspond to our observation that colchicine administration to mice not only reduces LPS clearance, but also increases LPS-induced blood concentrations of TNF-α and IL6.

In conclusion, intravital two-photon microscopy allowed us to image the process of particle and LPS phagocytosis by KC and its inhibition due to tubulin depolymerization.

## Supplementary Information

Below is the link to the electronic supplementary material.

Supplementary file1 (M4V 7326 KB)

Supplementary file2 (M4V 7352 KB)

Supplementary file3 (MP4 7424 KB)

Supplementary file4 (M4V 10158 KB)

Supplementary file5 (MP4 9207 KB)

Supplementary file6 (M4V 51597 KB)

Supplementary file7 (M4V 46408 KB)

Supplementary file8 (MP4 58988 KB)

Supplementary file9 (M4V 59845 KB)

Supplementary file10 (DOCX 14 KB)

## Abbreviations

ALT: Alanine transaminase
AST: Aspartate transaminase
AP: Alkaline phosphatase
IL6: Interleukin 6
i.p.: Intraperitoneal
KC: Kupffer cells
LPS: Lipopolysaccharide
TNF-α: Tumor necrosis factor alpha
