# Supplementary material for: Colchicine overdose impairs the capacity of Kupffer cells to clear foreign particles and endotoxins
Source: Arch Toxicol. 2022 Sep 14;96(11):3067–76. doi: 10.1007/s00204-022-03353-8 (PMC9525399; doi:10.1007/s00204-022-03353-8)
Supplement: Supplementary file 10 — Supplementary file10 (DOCX 14 KB) [file 204_2022_3353_MOESM10_ESM.docx]

**Supplemental video 1. Intravital imaging of Kupffer cells in control and in colchicine-intoxicated mice. A; B.** Kupffer cells of control mice visualized by intravenous administration of a red fluorescent F4/80 antibody (**A**) or by using the macrophage reporter mouse LysM (**B**) showing elongated morphology with many protrusions with hyperactive motility. **C.** Kupffer cells of colchicine-intoxicated mice (6 hours) visualized by intravenous administration of a red fluorescent F4/80 antibody showing roundish morphology with strongly reduced cell protrusion and motility. The videos correspond to Figure 4 of the main manuscript.

**Supplemental video 2. Compromised nanoparticle uptake capacity of Kupffer cells after colchicine intoxication. A.** Intravitally recorded video in a control mouse showing rapid phagocytosis of green-fluorescent, 100 nm-size, nanoparticles after intravenous administration. **B.** Intravitally recorded video 6 hours after colchicine intoxication showing failure of Kupffer cells to phagocytose the nanoparticles. The Kupffer cells are visualized by a red fluorescent F4/80 antibody. The videos correspond to Figure 5 of the main manuscript.

**Supplemental video 3. Intravital imaging of nanoparticle capture and internalization by Kupffer cells in control and colchicine-intoxicated mice. A.** Intravitally recorded video in a control mouse after intravenous administration of green-fluorescent 100 nm-size nanoparticles showing rapid capture of the nanoparticle aggregates (e.g., at 0.7-0.9 min) followed by the relatively slow uptake during which time the cell cytoplasm gradually becomes green. **B.** Intravitally recorded video 6 hours after colchicine intoxication showing failure of Kupffer cells to capture and internalize the nanoparticles. The Kupffer cells are visualized by a red fluorescent F4/80 antibody. The videos correspond to Figure 6 of the main manuscript.

**Supplemental video 4. Compromised LPS clearance by Kupffer cells after colchicine intoxication. A.** Intravitally recorded video in a control mouse showing rapid clearance of green-fluorescent LPS by Kupffer cells after intravenous administration. In addition, LPS is also slowly taken up by the sinusoidal endothelial cells and by hepatocytes and excreted in bile canaliculi. The position of the bile canaliculi is identified by the green-fluorescent bile acid analogue CLF administered intravenously approximately 15 minutes before the end of recording. **B.** Compromised LPS uptake by Kupffer cells and by hepatocytes after colchicine intoxication; in contrast LPS uptake by the sinusoidal endothelial cells was not affected by colchicine intoxication. The Kupffer cells are visualized by a red fluorescent F4/80 antibody. The videos correspond to Figure 7 of the main manuscript.
